# Comparison of physical examination and laboratory data between a clinical study and electronic health records

**DOI:** 10.1371/journal.pone.0236189

**Published:** 2020-07-22

**Authors:** Yi-An Ko, Yingtian Hu, Arshed A. Quyyumi, Lance A. Waller, Eberhard O. Voit, Thomas R. Ziegler, Michelle Lampl, Greg S. Martin

**Affiliations:** 1 Department of Biostatistics and Bioinformatics, Rollins School of Public Health, Emory University, Atlanta, Georgia, United States of America; 2 Division of Cardiology, School of Medicine, Emory University, Atlanta, Georgia, United States of America; 3 Department of Biomedical Engineering, Georgia Institute of Technology, Atlanta, Georgia, United States of America; 4 Division of Endocrinology, School of Medicine, Emory University, Atlanta, Georgia, United States of America; 5 Department of Anthropology, Emory University, Atlanta, Georgia, United States of America; 6 Division of Pulmonary, Allergy, Critical Care, and Sleep Medicine, School of Medicine, Emory University, Atlanta, Georgia, United States of America; International University of Health and Welfare, School of Medicine, JAPAN

## Abstract

Research based on secondary analysis of data stored in electronic health records (EHR) has gained popularity, but whether the data are consistent with those collected under a study setting is unknown. The objective is to assess the agreement between data obtained in a prospective study and routine-care data extracted retrospectively from the EHR. We compared the data collected in a longitudinal lifestyle intervention study with those recorded in the EHR system over 5 years. A total of 225 working adults were recruited at an academic institution between 2008–2012, whose EHR data were also available during the same time period. After aligning the participants’ study visit dates with their hospital encounter dates, data on blood pressure, body mass index (BMI), and laboratory measurements (including high-density lipoprotein (HDL), low-density lipoprotein (LDL), triglycerides, and total cholesterol) were compared via a paired t-test for equivalence with pre-specified margins. Summary statistics were used to compare smoking status and medication prescriptions. Overall, data were consistent between the two sources (i.e., BMI, smoking status, medication prescriptions), whereas some differences were found in cholesterol measurements (i.e., HDL and total cholesterol), possibly due to different lab assays and subject’s fasting status. In conclusion, some EHR data are fairly consistent with those collected in a clinical study, whereas others may require further examination. Researchers should evaluate the consistency and quality of EHR data and compare them with other sources of data when possible.

## Introduction

The infrastructure supporting electronic health records (EHRs) in the U.S. healthcare systems has expanded dramatically over the past decade. Secondary use of EHR data is appealing to the research community due to the reduced cost and time associated with data collection. Since the National Institutes of Health made leveraging EHRs for biomedical research a priority, researchers have been eagerly identifying and developing efficient methods for data accrual, integration, and analysis. For example, a growing number of large-scale biobanks have begun connecting dense, longitudinal EHR data with biorepositories among enrolled patients, thereby generating a new repository for medical research [1, 2].

While EHR data have great potential, a challenge is the assessment of their quality. It is commonly accepted that the quality and accuracy of the clinical data are not comparable to research standards due to differences in priorities between clinical and research settings [3]. Studies examining the validity of EHR data at the patient level, however, are rather limited and typically summarize inconsistencies between patient self-report and EHR documentation [4–7]. There is a need for more comprehensive evaluation of the utility, accuracy, and reliability of EHR data compared to more traditional sources of research data.

The present study seeks to assess the agreement between data obtained under a rigorous prospective study setting and prospective routine-care data for the same individuals, extracted from a university hospital data warehouse. The data elements included systolic blood pressure (SBP), body mass index (BMI), high-density lipoprotein cholesterol (HDL), low-density lipoprotein cholesterol (LDL), total cholesterol, and smoking status, and medication prescriptions.

## Materials and methods

### Study design

The investigation evaluated a longitudinal intervention study of generally healthy working adults without uncontrolled disease that focused on maintaining health under the auspices of the Emory/Georgia Tech Predictive Health Institute (Atlanta, GA, USA). The details of the study protocol has been described previously [8]. Briefly, a total of 711 Emory University or Healthcare employees were enrolled in 2008–2012. Each subject was assigned a health partner, who worked with the individual to establish a personalized action plan promoting a healthy lifestyle. Subjects were followed for five years, with visits at six and twelve months, followed by annual visits. During each visit, blood pressure (average of three measures) and BMI were measured and blood samples were taken to monitor health status. The study was approved by the institutional review board of Emory University. Written informed consent was obtained from all participants.

**Fig 1:**
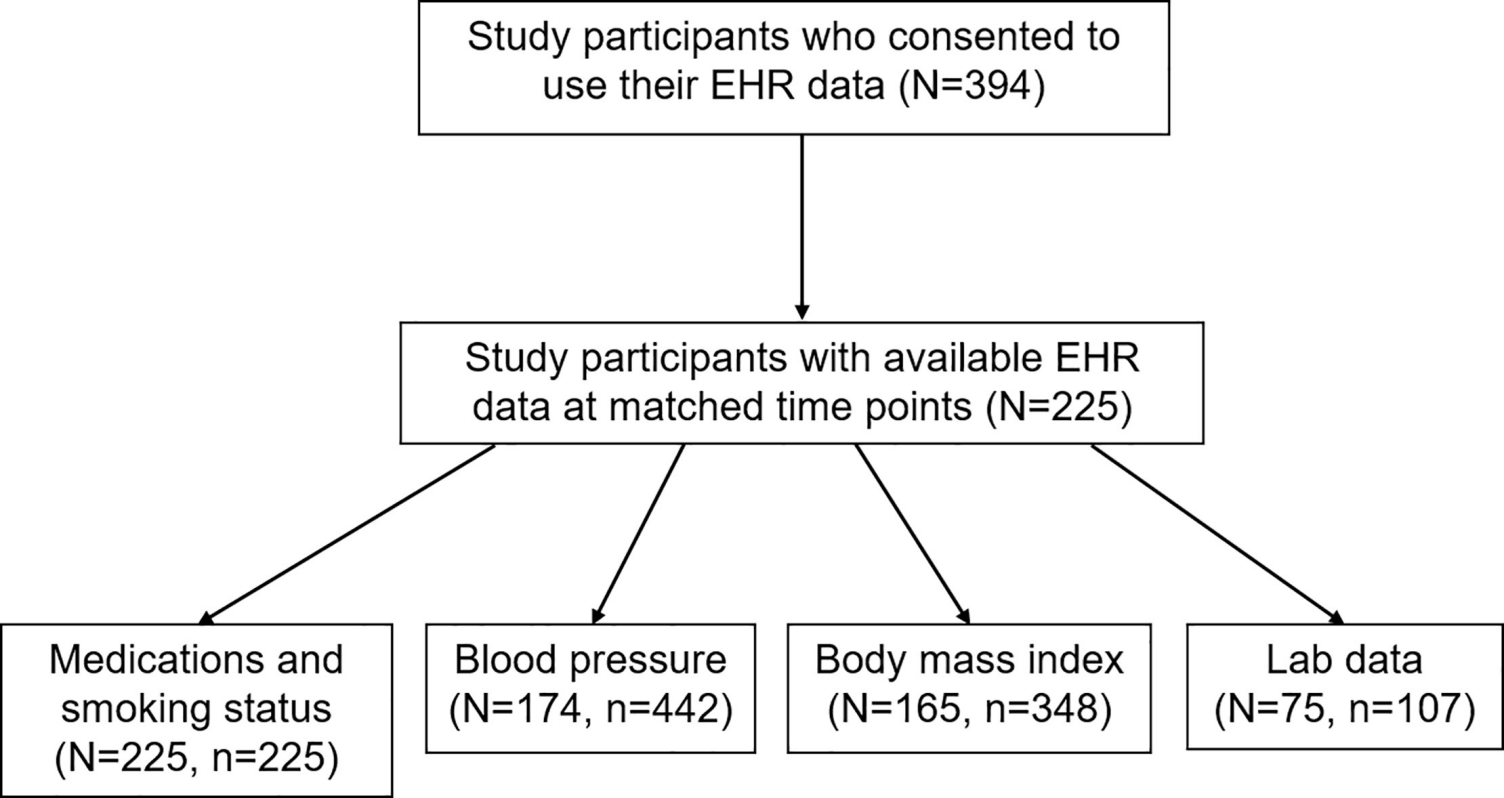
Numbers of participants (N) and numbers of observations (n) in various comparisons between standard clinical measurements an EHR data.

A total of 394 consented to the use of their EHR data for researches, and data of 225 were electronically available. We aligned the time points of measurements from the two data sources such that the baseline hospital visit fell within 90 days prior to the study baseline visit, to avoid any potential intervention effect. For each follow-up visit, EHR data had been obtained within +/- 45 days of the study visit. Fig 1 shows numbers of subjects with available data for SBP, BMI, and lipid profiles after this data alignment procedure. All the subjects were fasting during study visits and their blood samples were analyzed by Quest Diagnostics Lipid Panel, but fasting was not required during Emory hospital visits and the lipid profiles were tested using Beckman Coulter AU5800 analyzers [9]. Medication use at baseline visit were obtained by prescription data (EHR) and self-report questionnaires (clinical study). The medication data were categorized into yes/no for hypertension, diabetes control, and lipid lowering by carefully reviewing individual medication names by two cardiologists. For example, “Avapro” was categorized as hypertension and diabetes medication, “Simvastatin” was categorized as lipid lowering medication.

### Statistical analysis

The distributions of variables of interest were compared using kernel density plots and counts (percentages). To compare SBP, BMI, and lipid measurements, concordance correlation coefficient (CCC) and Bland–Altman plots were used to estimate the overall concordance and agreement between the two data sources. Paired t-tests for equivalence using two one-sided tests procedure [10, 11] were conducted by setting the margins at 5 mmHg for SBP, 0.5 kg/m^2^ for BMI, 5 mg/dL for total cholesterol, triglycerides, and LDL, and 2 mg/dL for HDL accounting for natural day-to-day variations [12, 13]. Cohen's kappa (κ) was calculated for medications and smoking data. R 3.6.0 was used for analysis, and the significance level was 0.05.

## Results

Among the 225 consented participants available in the EHR, the mean (SD) age of participants was 50 (9.6) years (age range 24–77 years); 59% were female, and 41% were black. The characteristics of the two subgroups with SBP and BMI data were similar to the 225 participants, while those with lab data (N = 75) were 3 years older on average, and only 29% were blacks.

**Fig 2:**
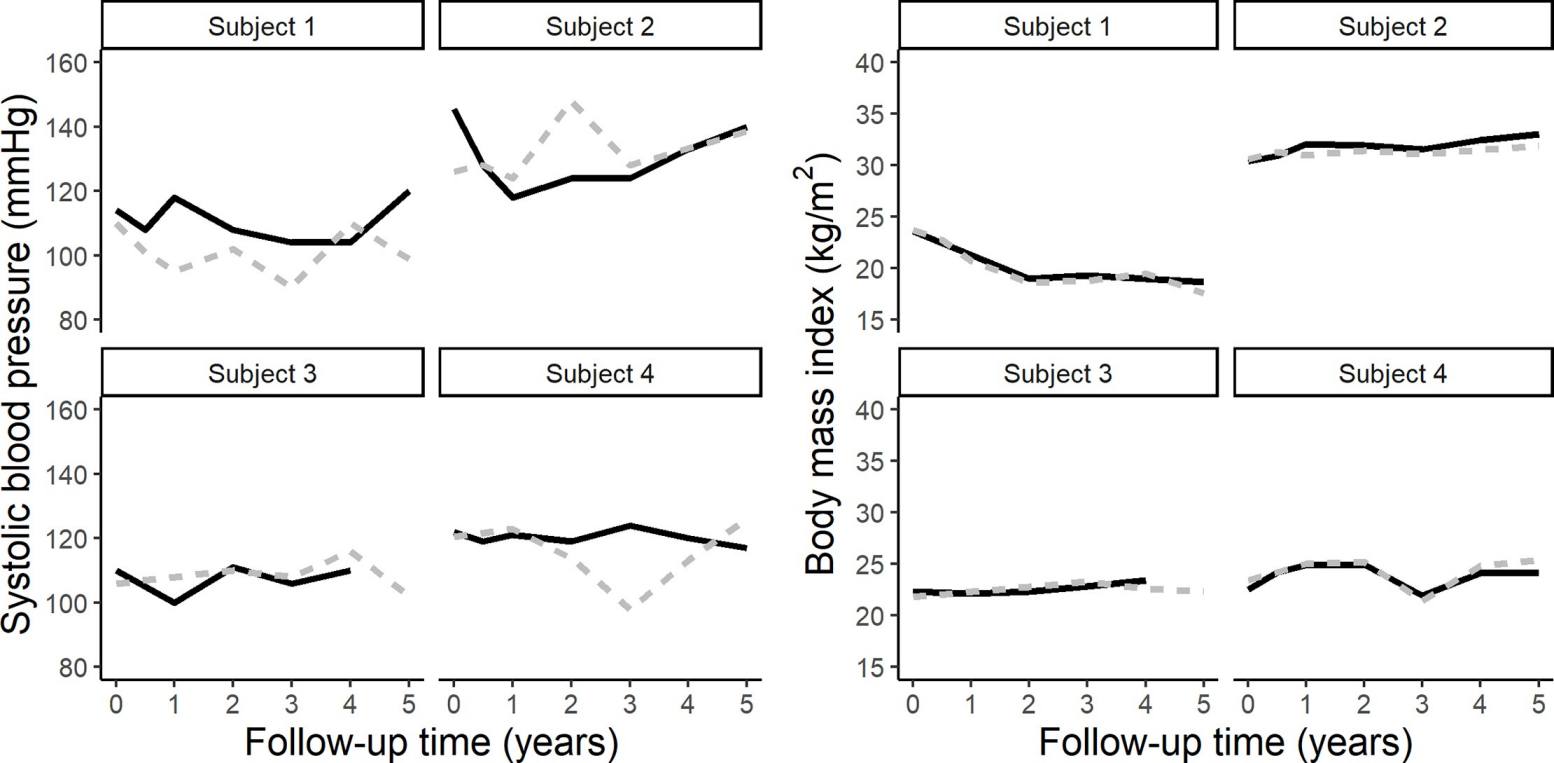
Examples of systolic blood pressure (a) and body mass index measurements (b) of 4 subjects, obtained from the Predictive Health clinical study (solid line) and the EHR system (dashed line) over five years.

**Fig 3:**
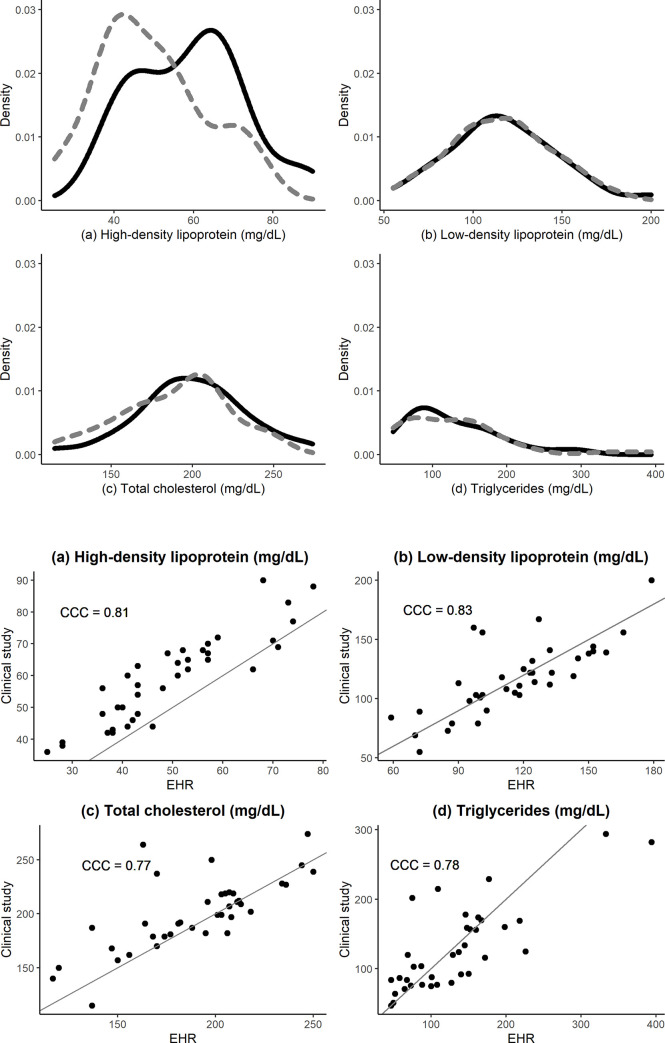
Baseline distributions of high-density lipoprotein (a), low-density lipoprotein (b), total cholesterol (c), and triglycerides (d) measurements from the clinical study (solid line) and the EHR system (dashed line).

Fig 2 shows the individual-level trajectories of SBP and BMI over time from four randomly selected participants, and the distributions of data from the two sources. Although the trajectories of SBP did not entirely overlap, the mean difference in SBP across all repeated measurements was 0.62 mmHg (95% CI = [-0.60, 1.83]; CCC: 0.54). BMI appeared to be a robust measure with a mean difference of 0.20 kg/m^2^ (95% CI = [0.12, 0.29]; CCC: 0.98). Fig 3 shows the density and scatter plots of HDL, LDL, triglycerides, and total cholesterol baseline measurements. The total cholesterol and triglycerides distributions appear to be somewhat different. After accounting for all the repeated measurements, the clinical data indicated significantly higher HDL concentrations than the EHR data, with a mean difference of 6.76 mg/dL (95% CI = [5.35, 8.18]; CCC: 0.81). Similarly, the mean of total cholesterol was 9.26 mg/dL (95% CI = [5.27, 13.25]; CCC: 0.77) higher in the clinical data vs. EHR. Nevertheless, both triglycerides and LDL measurements were similar with mean differences of 1.46 mg/dL (95% CI = [-7.11, 10.03]; CCC: 0.78) and 1.42 mg/dL, 95% CI = [-1.71, 4.55], CCC: 0.83), respectively.

Additionally, medications for hypertension, diabetes, and dyslipidemia as well as smoking status at study baseline, were compared. Self-reported medications were compared with prescriptions documented in the EHR. Among the 225 participants, 206 (92%), 222 (99%), and 216 (95%) had consistent medication use for hypertension (κ = 0.74), diabetes (κ = 0.84), and dyslipidemia (κ = 0.87), respectively. Specifically, the percentages of under- and over-reporting of hypertension and diabetes medications were equal in the clinical study. Nine people prescribed with statins (according to their EHR) did not report them. Smoking status was consistent in 95% of the comparisons, with 10 participants reporting smoking in the study but non-smoking in the EHR, and 2 reporting non-smoking in the study but smoking in the EHR.

## Discussion

We demonstrated that, in general, measurements of BMI, and medication prescription/use in the study setting are by and large consistent with those documented in the EHR system. Given that measurements of blood pressure can be affected by various factors, they may not be comparable between the two data sources if one wishes to focus on individual-level data. When blood pressure measurements are handled in an aggregated form (e.g., mean difference), which is common for studies using EHR data, our findings suggest that EHR data are considerably consistent with the clinical data. This is encouraging for researchers who leverage existing EHR data in their studies. On the other hand, there appears to be a certain degree of discrepancy in lipid measurements, specifically with respect to lower levels of HDL and total cholesterol observed in the EHR system.

Several reasons possibly account for these observed differences. The differences in blood pressure could be due to different numbers of measures and resting status. Blood pressure was typically obtained by one single measure during clinic visit, whereas during the study visit, it was an average of 3 measures after relaxation. The inconsistencies in lipid component measurements could be due to differences in measurement time (diurnal variation), fasting status, and lab assays. A study investigated within-person variation in serum lipids reported a geometric mean of the within-person standard deviation of 5 mg/dL for total cholesterol and 1.5 mg/dL for HDL cholesterol with a median of 4 days blood collection, and the variation increased as collection interval increased [13]. Another study examined changes in lipids after meal compared with fasting in the general population and found a mean change of 3.6 mg/dL in total cholesterol and triglycerides [14]. Considering the magnitude of fluctuations reported in previous studies and that in our current study the EHR data were extracted within +/- 45 days of the study visit date, our observed differences between the clinical study and the EHR data may be reasonable. In addition, the fact that lipid cholesterol measured by two different lab assays could be responsible for the differences. Lastly, the self-reported information on medication use and EHR prescription data had satisfactory agreement, which was similar to a study using pharmacy records [15]. When there are discrepancies between the two data sources, the EHR mediation prescriptions should be accurate given potential recall bias in the self-reported medication use data.

Although there have been a number of studies assessing the consistency between a patient’s medical record and self-reported data [4–7, 16]. our study is the first that examines the agreement between data obtained under a rigorous study setting versus those obtained from EHR data extraction. The findings imply caution when comparing and aligning data across different sources. One limitation of our study is the modest sample size after aligning data from two sources, especially the laboratory data. Also, our data were obtained from a single center, which may not be generalized to other institutions or places. Future work should investigate the reasons of inconsistencies in lipid measurements and expand the study to explore other data types.

## References

[1] Denaxas SC, George J, Herrett E, Shah AD, Kalra D, Hingorani AD, et al Data Resource Profile: Cardiovascular disease research using linked bespoke studies and electronic health records (CALIBER). Int J Epidemiol. 2012;41(6):1625–38. 10.1093/ije/dys188 23220717 PMC3535749

[2] Kerr SM, Campbell A, Marten J, Vitart V, McIntosh AM, Porteous DJ, et al Electronic health record and genome-wide genetic data in Generation Scotland participants. Wellcome Open Res. 2017;2:85 10.12688/wellcomeopenres.12600.1 29062915 PMC5645708

[3] Weiner MG, Embi PJ. Toward reuse of clinical data for research and quality improvement: the end of the beginning? Ann Intern Med. 2009;151(5):359–60. 10.7326/0003-4819-151-5-200909010-00141 19638404

[4] Valikodath NG, Newman-Casey PA, Lee PP, Musch DC, Niziol LM, Woodward MA. Agreement of Ocular Symptom Reporting Between Patient-Reported Outcomes and Medical Records. JAMA Ophthalmology. 2017;135(3):225 10.1001/jamaophthalmol.2016.5551 28125754 PMC5404734

[5] Rodriguez HP, Glenn BA, Olmos TT, Krist AH, Shimada SL, Kessler R, et al Real-World Implementation and Outcomes of Health Behavior and Mental Health Assessment. J Am Board Fam Med. 2014;27(3):356–66. 10.3122/jabfm.2014.03.130264 PMC4237013 24808114

[6] Rolnick SJ, Parker ED, Nordin JD, Hedblom BD, Wei F, Kerby T, et al Self-report compared to electronic medical record across eight adult vaccines: do results vary by demographic factors? Vaccine. 2013;31(37):3928–35. 10.1016/j.vaccine.2013.06.041 23806243 PMC4689428

[7] Tisnado DM, Adams JL, Liu H, Damberg CL, Chen WP, Hu FA, et al What is the concordance between the medical record and patient self-report as data sources for ambulatory care? Med Care. 2006;44(2):132–40. 10.1097/01.mlr.0000196952.15921.bf 16434912

[8] Al Mheid I, Kelli HM, Ko YA, Hammadah M, Ahmed H, Hayek S, et al Effects of a Health-Partner Intervention on Cardiovascular Risk. J Am Heart Assoc. 2016;5(10). 10.1161/JAHA.116.004217 PMC5121518 27729334

[9] Nikolac Gabaj N, Miler M, Vrtaric A, Hemar M, Filipi P, Kocijancic M, et al Precision, accuracy, cross reactivity and comparability of serum indices measurement on Abbott Architect c8000, Beckman Coulter AU5800 and Roche Cobas 6000 c501 clinical chemistry analyzers. Clin Chem Lab Med. 2018;56(5):776–88. 10.1515/cclm-2017-0889 29315074

[10] Lakens D, Scheel AM, Isager PM. Equivalence Testing for Psychological Research: A Tutorial. Advances in Methods and Practices in Psychological Science. 2018;1(2):259–69.

[11] Schuirmann DJ. A comparison of the two one-sided tests procedure and the power approach for assessing the equivalence of average bioavailability. J Pharmacokinet Biopharm. 1987;15(6):657–80. 10.1007/BF01068419 3450848

[12] Morris CJ, Hastings JA, Boyd K, Krainski F, Perhonen MA, Scheer FA, et al Day/night variability in blood pressure: influence of posture and physical activity. Am J Hypertens. 2013;26(6):822–8. 10.1093/ajh/hpt026 23535155 PMC3693479

[13] Pereira MA, Weggemans RM, Jacobs DR Jr., Hannan PJ, Zock PL, Ordovas JM, et al Within-person variation in serum lipids: implications for clinical trials. Int J Epidemiol. 2004;33(3):534–41. 10.1093/ije/dyh057 15020568

[14] Langsted A, Freiberg JJ, Nordestgaard BG. Fasting and nonfasting lipid levels: influence of normal food intake on lipids, lipoproteins, apolipoproteins, and cardiovascular risk prediction. Circulation. 2008;118(20):2047–56. 10.1161/CIRCULATIONAHA.108.804146 18955664

[15] Drieling RL, LaCroix AZ, Beresford SAA, Boudreau DM, Kooperberg C, Heckbert SR. Validity of Self-Reported Medication Use Compared With Pharmacy Records in a Cohort of Older Women: Findings From the Women's Health Initiative. Am J Epidemiol. 2016;184(3):233–8. 10.1093/aje/kwv446 27402774 PMC4967595

[16] Wagaw F, Okoro CA, Kim S, Park J, Rachman F. Linking Data From Health Surveys and Electronic Health Records: A Demonstration Project in Two Chicago Health Center Clinics. Prev Chronic Dis. 2018;15:E09 10.5888/pcd15.170085 29346063 PMC5774304

